# Resistance to bio-insecticides or how to enhance their sustainability: a review

**DOI:** 10.3389/fpls.2015.00381

**Published:** 2015-06-19

**Authors:** Myriam Siegwart, Benoit Graillot, Christine Blachere Lopez, Samantha Besse, Marc Bardin, Philippe C. Nicot, Miguel Lopez-Ferber

**Affiliations:** ^1^Institut National de la Recherche Agronomique, UR1115, Plantes et Systèmes de Culture Horticoles UnitAvignon, France; ^2^Laboratoire de Génie de l'Environnement Industriel, Ecole des Mines d'Alès, Institut Mines-Telecom et Université de Montpellier Sud de FranceAlès, France; ^3^Natural Plant Protection, Arysta LifeScience GroupPau, France; ^4^Institut National de la Recherche AgronomiqueAlès, France; ^5^Institut National de la Recherche Agronomique, UR407, Plant Pathology UnitMontfavet, France

**Keywords:** sustainability, mode of action, Bt, CpGV, mechanism of resistance, molecular target mutation, detoxification enzymes, efficacy

## Abstract

After more than 70 years of chemical pesticide use, modern agriculture is increasingly using biological control products. Resistances to conventional insecticides are wide spread, while those to bio-insecticides have raised less attention, and resistance management is frequently neglected. However, a good knowledge of the limitations of a new technique often provides greater sustainability. In this review, we compile cases of resistance to widely used bio-insecticides and describe the associated resistance mechanisms. This overview shows that all widely used bio-insecticides ultimately select resistant individuals. For example, at least 27 species of insects have been described as resistant to *Bacillus thuringiensis* toxins. The resistance mechanisms are at least as diverse as those that are involved in resistance to chemical insecticides, some of them being common to bio-insecticides and chemical insecticides. This analysis highlights the specific properties of bio-insecticides that the scientific community should use to provide a better sustainability of these products.

## Introduction

The awareness of secondary effects of pesticide use on the environment and human health is currently causing a green revolution. Modern agriculture is changing; it tends to spread less pesticide and generally use more-selective and less-polluting products. Bio-pesticides fit this definition, and in recent years, pesticide firms have invested significantly in companies producing bio-pesticides.

Bio-pesticides have a reputation of being very expensive and often not reliable. To date, the price of bio-pesticides is still high because of their low market share compared to the chemical compounds. The market size of bio-pesticides increased 9.9% between 2005 and 2010. In the same period, the market size of synthetic pesticides decreased by 1.5% (Tanwar et al., 2012). For all crop types, bacterial bio-pesticides represent 74% of the market, fungal bio-pesticides represent approximately 10%, viral bio-pesticides 5%, predator bio-pesticides 8% and other bio-pesticides 3% (Thakore, 2006). In the 1990s, products based on *Bacillus thuringiensis* (*Bt*) accounted for 95% of the global microbial insecticide market. Currently, the apparition of new biological control agents, such as beneficial insects, viruses and entomopathogenic fungi, has lowered the prevalence of *Bt*, which still represents more than 60% of the bio-pesticide market. The panel of biological agents that are available on the market is gradually increasing and should continue to increase over the next few years due to their variety (not only microorganisms but also predators and parasites).

One of the major issues in bio-pesticide development is their lack of robustness and the high-tech level that is necessary for their utilization (Ravensberg, 2011). Indeed, these products are generally more sensitive to variations in temperature, humidity or light radiation than are their synthetic counterparts. Formulation technologies have been used to improve the number of commercial biological control products, the delivery, the shelf life and the field efficacy of bio-pesticides by adding various compounds to the active agents (Leggett et al., 2011; Ravensberg, 2011). Although there is limited information on the research in this area, due to concurrent issues, recent studies have permitted the emergence of (i) new formulations (Townsend et al., 2004; Hunter, 2010) (ii) formulations with precise time or location delivery (Lacey, 2007; Nuyttens et al., 2009), and (iii) formulations helping with long activity persistence (Kohl et al., 1998).

However, the shorter persistence of bio pesticides can be considered a quality as it reduces their environmental risk, but this often results in the more frequent pulverization of those products to maintain an acceptable level of control. The quantity of products that are released into the environment is therefore more important, and the costs of these multiple interventions make biological control less competitive than chemical control. Moreover, the maintenance of selection pressures and the relatively low efficiency levels (70%) of biocontrol products favor resistance development. In recent years, many studies have been conducted to improve the efficiency, ease of use and persistence of these products.

The possible side effects of the use of bio-pesticides have often been neglected or minimized compared to those of chemical pesticides (Holt and Hochberg, 1997), and cases of resistance have been described. Thus, the question of the sustainability of these strategies over time arises. At least 27 pest species have developed resistance to the most used bio-pesticide in the world: *Bacillus thuringiensis* (Berling et al., 2009; Bravo et al., 2011).

We propose in this review a detailed inventory of cases of resistance to bio-pesticides to understand this phenomenon and anticipate it better. We rely on experience gained from numerous studies on resistance to chemical insecticides and the best ways to manage it to propose new solutions thanks to the intrinsic properties of bio-insecticides. To investigate this problem, we decided to present modes of action and resistance mechanisms for the main bio-insecticides. Then, we evaluated the durability of biocontrol agent efficacy to determine the risks factors of resistance against future biocontrol agents.

Bio-pesticide is a term that was first applied with a narrow focus on preparations containing living microorganisms and then expanded to a broader definition that encompasses botanical compounds and semiochemicals (e.g., pheromones) (Kiewnick, 2007). In this review, bio-pesticide will include botanical insecticides (plant extracts), microbial pest control agents (bacteria, viruses, fungi and protozoa) and/or bioactive compounds (such as metabolites) that are produced directly from these microbes and used to suppress populations of insect pests.

In addition, because the aim of this review is to provide a critical point of view on the sustainability and effectiveness of bio-pesticides, we present some examples of commercialized and currently used products (Genetically modified (GMO) crops and human disease vectors excluded).

## Main mode of action of bio-control agents

### Plant extracts (botanical insecticides)

To control their natural enemies, some plants have developed the ability to synthesize products that are derived from their secondary metabolism with some specific properties against insects (Isman, 2006). Those molecules may be repulsive, cause metabolic dysfunctions, or have a toxic effect leading to the death of the insect, which is the case of caffeine in coffee, glucosinolates in *Brassicaceae*, psoralen in celery or even nicotine in tobacco.

Insecticides that are derived from plants have been used for centuries. In the 17th century, decoctions of tobacco, containing nicotine, were applied to some crops. These natural insecticides experienced significant development between the two world wars before being progressively overtaken by synthetic insecticides, which are cheaper and more efficient.

Among the variety of natural molecules having insecticidal properties, we focus on the most frequently used, such as alkaloids, including nicotine and pyrethrums.

#### Nicotine

Nicotine is a molecule that is extracted from the leaves and stems of tobacco *Nicotiana tabacum*. The use of nicotine dates back to 1690 when aqueous extracts of tobacco were used to control crop pests. This molecule is very stable and has an important toxicity for insects. However, as nicotine is also toxic for mammal species, its use is limited. The use of nicotine is still authorized in some countries as a complex preparation (in alkaline sulfate solution or in soaps). This alkaloid affects the insect by inhalation, ingestion and contact. The target of nicotine is the nicotinic acetylcholine receptor (nAChRs) of the central nervous system. The fixation of nicotine, an acetylcholine mimetic, on the postsynaptic receptor is responsible for the continuous depolarization of nerve cells, leading to a permanent excitation. This excitation leads to the muscular paralysis of the insect and to its death (Bai et al., 1991). Based on this mode of action, synthetic neonicotinoids have been developed, avoiding some of the problems of toxicity to mammals.

#### Pyrethrum

Pyrethrum is a botanical insecticide that is allowed in organic farming in many countries worldwide. This compound is extracted from dried *Chrysanthemum* flowers, especially from *Dalmatia pyrethrum*. Theses flowers contain some insecticidal products, such as a group of esters, including “pyrethrins,” which harbor the highest relative toxicity. Pyrethrins are very unstable compounds that are quickly degraded by light, air and heat. To reinforce the action of the pyrethrum, some synergistic molecules can be added to the formulation; the main molecule is piperonylbutoxide (PBO). Pyrethrum containing some PBO is allowed in organic farming but is subject to controversy due to its toxicity (Jansen et al., 2010).

Pyrethrins attack the nervous system of insects. These compounds impact the sodium channels by blocking them in an open position by the inhibition of the voltage-dependent inactivation (Casida, 1973). The result of this blockage is a significant release of acetylcholine at the synapse level. This over-abundance of neurotransmitters causes the desensitization of postsynaptic receptors and then triggers, through negative feedback on the pre-synaptic receptors, an inhibition of acetylcholine release. The synaptic transmission is then blocked, which leads to the paralysis of the insect followed by its death.

#### Neem oil

Neem oil is extracted from the neem seeds of *Azadirachta indica*, a tree originating from India. This tree grows in poor and degraded soils and in semi-arid climates. The oil that is extracted from these seeds has a repellent, antifeedant and insecticide power. More than hundred molecules having insecticidal and/or repellent activities have been identified in the crude oil, most belonging to the family of terpenoids. The principal active substance of this oil is azadirachtin (Akhila and Rani, 2002). Both the crude neem oil and the purified azadirachtin are used in pest control. Neem oil can be home-made or industrially produced, and its composition is variable. Neem oil can be used crude with its entire cocktail of molecules or after purification to retain only azadirachtin.

Azadirachtin is soluble in polar solvents; this is a complex molecule whose structure has been difficult to determine (Broughton et al., 1986; Bilton et al., 1987; Taylor, 1987; Turner et al., 1987). This molecule, which unstable to light, has a repellent, antifeedant and growth-destabilizing power on the insects that are not repelled. According to Ruscoe (1972), Morgan (2009), the insecticidal power of azadirachtin is effective at a far lower dose than its repellent power. This molecule has been tested on more than 600 insects, among which more than 500 are sensitive (Morgan, 2009). It seems that a synergy exists between the different compounds of unpurified neem oil. In the field, neem oil is used as a fertilizer, plant natural stimulator, insecticide, fungicide and pest repulsive. The insecticidal activity of this oil is also used in animal breeding, for the disinfection of livestock buildings or directly applied to animals to prevent some parasites, such as ticks and fleas.

The first mode of action known for neem oil is its repellent/anti-feedant power, partly due to azadirachtin. Without finding a specific target, it has been hypothesized that azadirachtin has a mainly anti-feedant action (Simmonds et al., 1990; Nisbet et al., 1993; Koul et al., 2004).

However, the neem oil was later shown to exhibit a complex growth-disturbing action. Some histologic studies have been carried out on the neurosecretions of peptide hormones in insect brains. These studies reveal a dysfunction in the synthesis, transport and secretion of three hormones: the prothoracicotropic hormone (PTTH), the allatostatins and the allatoinhibins. These hormones play a role in the synthesis and release of mating hormones: ecdysteroids and juvenile hormone (Subrahmanyam et al., 1989; Subrahmanyam and Rembold, 1989; Sayah et al., 1996, 1998). Insects are disrupted during their growth and mate. Reproductive functions can also be affected by these hormonal changes. In females, there is a degeneration of ovaries and a breakdown of the egg yolk (Koul, 1984; Schmutterer, 1985; Dorn et al., 1987). In males, spermatogenesis is interrupted before metaphase. However, the two primary effects of azadirachtin seem to be the blockage of calcium channels (Qiao et al., 2014) and the mitochondria-mediated apoptosis of cells (Huang et al., 2013). These two actions induce a cascade of effects, such as the blockage of mitosis (Huang et al., 2011) and a reduction in protein synthesis (Timmins and Reynolds, 1992; Koul et al., 1996).

### Bacteria and derivates

There are more than 100 bacterial species having entomopathogenic activity (Starnes et al., 1993), and the species *Bacillus thuringiensis*, *B. popilliae*, *B. lontimorbus*, *B. sphaericus*, and *Saccharopolyspora spinosa* are the most used.

#### Bacillus thuringiensis

*Bacillus thuringiensis* (*Bt*) is the most used and described bio-insecticide in the world. This species is an aerobic bacterium, Gram-positive, of the bacilli family and is widely present in soil, water, air and plants in its vegetative form, although it is not clear how important its multiplication is in these environments (Raymond et al., 2010). When the trophic environment is favorable, this species multiplies by binary fission and lives in a vegetative form. Under deficient trophic conditions, this species takes a sporulated form, producing proteins that are toxic for insects. Among these proteins, the Cry and Cyt proteins form parasporal crystals (Bravo et al., 2011). Since the 1930s, the entomotoxic properties of this bacterium have raised an agricultural interest, which has been materialized by its first usage in 1933. This bacterium has been used since the 1950s. First against defoliating Lepidoptera in 1970, the discovery of serotypes active against *Diptera* (1977) and *Coleoptera* (1981) has permitted the extension of the larvicidal action of this species to mosquitoes, black-flies, and beetles. Many different isolates of *Bt* have been described and are classified by serology and by the targets of their toxin proteins (Lecadet et al., 1999). The toxins are classified according to their amino acid sequence (Crickmore et al., 1998). The most commonly used bacterial serovars are *kurstaki* against defoliating Lepidoptera larvae, *aizawai* against Lepidoptera larvae feeding on seed and *san diego* and *tenebrionis* (= *morrisoni*) against *Coleoptera* larvae. An additional commonly used serovar, *Bt* var. *israeliensis*, is used against mosquito vectors of human diseases (Bravo et al., 2011). Commercialized *Bt* products are made of a mixture of spores and protein crystals (Raymond et al., 2010) and represented 2% of the insecticides on the market in 2011 (Bravo et al., 2011).

There is a great variety of Cry toxins that belong to the class of PFT (Pore-Forming Toxins) (Pigott and Ellar, 2007). The three-dimensional structure and detailed mode of action have been the object of important studies (see Bravo et al., 2011, for review). The final outcome of toxin ingestion is the destruction of the insect midgut via the formation of pores in the midgut cells. The body content becomes accessible to bacterium spores that develop in this rich culture medium.

The result is a septicemia that is caused not only by *Bt* but also by other bacterial species (Raymond et al., 2010). The insect dies because it is not able to regenerate new epithelial cells on time. Death is slow in the absence of bacteria and rapid in the case of septicemia. Other marginal effects are observed, such as a decline in growth or development delay (Heckel, 2012).

The transmission of *Bt* is mainly horizontal, either from one contaminated larva to another or via the plants that contain the bacterium (Raymond et al., 2010).

#### Spinosyns

*Saccharopolyspora spinosa* is an actinomycete (Gram-positive bacterium) that was discovered in 1982 in a soil sample from the Virgin Islands. Molecules with insecticidal activity (spinosyns) are extracted from the bacterium after fermentation (Sparks et al., 1998). Today, these molecules are mainly commercialized as two active substances: the spinosad and the spinetoram.

Spinosad is a mixture of spinosyns A and D, the two most active metabolites that are produced by the species. This product was first approved in the United States in 1997 to control Lepidoptera larvae that were resistant to pyrethroids. This insecticide has been used for many years on a considerable number of pests worldwide. Today, this insecticide is used in 40 countries on various crops, such as cotton, crucifer, apple, grapevine and peach, to control more than 50 pests. In 2007, Spinetoram (mixture of two: J “major” and L “minor”) was approved in the United States and in Canada after clear evidence of resistance to spinosad among various pests in both the laboratory and the field (Sparks et al., 2012). Once ingested by the insect, spinosyns quickly reach the central nervous system, where they induce the depolarization of the neuron membranes that are connected to the muscles. This hyperexcitation causes insect paralysis. Spinosyns fix at a specific site on the acetylcholine receptor (nAchR) that are different from the neonicotinoid sites. These compounds also act on the GABA (γ-aminobutyric) receptors, but their functions are not yet clearly defined (Sparks et al., 2012).

#### Other bacteria

In the case of *Bacillus sphaericus*, the toxin is located on the spore wall and is released by the partial digestion of the bacteria in the insect larvae digestive track. The toxin passes through the peritrophic membrane of the digestive track and kills the larvae (Singer, 1981; Burges, 1982). The bacterium multiplies in the host and is released by its disintegration. Since their introduction on the market to control *Diptera*, many cases of resistance to the crystal toxin have been reported (Miller et al., 1983; Charles et al., 1996).

### Fungus

Entomopathogenic fungi are known and studied for their insecticidal properties. To date, there are more than 700 species listed (Hajek and Stleger, 1994). Among these fungi, nine species are commercialized or regularly studied: *Beauveria bassiana*, *B. brongniartii*, *Metarhizium anisopliae*, *Aschersonia aleyrodis*, *Lecanicillium* (*Verticillium*) *lecanii*, *Paecilomyces fumosoroseus*, *Entomophaga maimaiga*, *Hirsutella thompsonii*, and *Lagenidium giganteum*.

These fungi act as hyperparasites and penetrate into their host through natural breaches in the cuticle or by creating breaches with enzymes, such as chitinases. Chitinases are virulent determinants and are essential for pathogenicity, for example, in *B. bassiana* (Fang et al., 2005) and *M. anisopliae* (Prakash et al., 2012).

Other insect-wall-degrading enzymes are synthesized by these entomopathogenic fungi, for example, cuticle-degrading protease, which is classified into two families, Pr1 and Pr2 (Castellanos-Moguel et al., 2007). In *M. anisopliae*, a Pr1 causes the melanization of the insect, which is a normal immune response in insects, but in extreme cases, it can lead to insect death (St Leger et al., 1996).

### Viruses

Several virus families have been identified for their ability to infect arthropods; however, Baculoviruses are the only ones that are used as biological control agents in practice (Table 1) (Dent, 1991; Suty, 2010).

**Table 1:** **Commercialized bio-pesticides with baculoviruses (Berling, 2009; Eberle et al., 2012; Yang et al., 2012; Beas-Catena et al., 2014)**.

**Virus**	**Pest**	**Crop**	**Country**
AdorGV	*Adoxophyes orana*	Orchards	Switzerland
AsGV	*Agrotis segetum*	Vegetables	Hungary
ClGV	*Cryptophlebia leucotreta*	Orchards	Switzerland
CpGV	*Cydia pomonella*	Orchards	France
			Argentina
			USA
			Belgium
			Switzerland
			Canada
			Spain
MyseGV	*Mythimna separata*	Rice	China
PhopGV	*Phthorimaea operculella*	Potatoes	Colombia
	*Tecia solanivora (Symmestrischema tangolias)*		Bolivia
PiraGV	*Pieris rapeae*	Vegetable	China (one producer)
PiGV	*Plodia interpunctella*	Stored raisins and almond	USA
PoGV	*Phthorimaea operculella*	Field and stored potatoes	Peru
PxGV	*Plutella xylostella*	Vegetables, wheat, corn	China
AgMNPV	*Anticarsia gemmatalis*	Major crops	Brazil
AcMNPV	*Autographa californica*, *Estigmene acrea*	Major crops	Guatemala
	*Heliothis virescens*, *Plutella xylostella*	Vegetables	China (three producers)
	*Pseudoplusia includens*, *Spodoptera exigua*,		Guatemala
	*Trichoplusia ni*		
AfNPV	*Anagrapha falcifera*	Vegetable	USA
AgMNPV	*Anticarsia gemmatalis*	Soybean	Brazil
			Argentina, Mexico
BusuNPV	*Buzura suppressaria*	Tea	China (one producer)
EcobNPV	*Ectropis oblica pulina*	Tea	China (one producer)
EupsNPV	*Euproctis pseudoconspersa*	Tea	China
GyruNPV	*Gynaephora spp*	Pasture	China (one producer)
HearNPV	*Helicoverpa armigera*	Cotton, tomato	India
		Pepper, tobacco	Switzerland
			Australia
			China, Vietnam, Thailand
HzNPV	*Helicoverpa spp*.	Cotton, vegetables	USA
			Australia
HycuNPV	*Hyphantria cunea*	Forest	Moldavia, Russia
LeseNPV	*Leucania separata*	Wheat, maize	China (one producer)
LdNPV	*Lymantria dispar*	Forest	Canada
			USA
MabrNPV	*Mamestra brassicae*	Vegetables	USA and Japan
			Russia
MacoNPV	*Mamestra configurata*		Switzerland
MyseNPV	*Mythimna separata*	Rice	China
NeabNPV	*Neodiprion abietis*	Forest	Canada
NeleNPV	*Neodiprion lecontei*	Forest, ornamentals	Canada
	*Neodiprion sertifer*	Forest	
OpMNPV	*Orgyia leucostigma*	Forest	Canada
	*Orgyia pseudotsugata*	Ornamentals	
SeMNPV	*S exigua*	Major crops	Switzerland
	*Spodoptera littoralis*	Vegetables	India
			USA
			Spain
			China
SaMNPV	*Diaphania hyalinata, Spodoptera albula, S exigua, S sunia*	Major crops, vegetables	Guatemala
SpliNPV	*S littoralis*	Major crops, vegetables	Switzerland
SpltNPV	*S litura*	Cotton, vegetables, rice	India
			Vietnam

*Some of the mentioned products are locally made and do not have a commercial name. This list is not exhaustive*.

Baculoviruses form a large family of viruses that can infect Lepidoptera larvae crop pests, as well as *Hymenoptera, Diptera, Coleoptera and Trichoptera*. These species are relatively specific by infecting only one or a few closely related insect species. An exception is *Autographa californica* nucleopolyhedrovirus, AcMNPV, which infects more than 33 species from 7 families of Lepidoptera (Gröner, 1986; Bishop et al., 1995). These viruses replicate in the nucleus of the host cells. Their genome is composed of a double-stranded circular DNA molecule with a size varying from 80 to 200 kb. The genome is packed into a nucleocapsid presenting helicoidal symmetry that is enveloped in a membrane. Baculoviruses present a complex life cycle in which two viral forms are produced (see Rohrmann, 2013, for a review).

Baculoviruses are classified into four genera according to the International Committee on the Taxonomy of Viruses (release 2013, EC45): the Alphabaculoviruses, (nucleopolyhedroviruses (NPV) infecting Lepidoptera), Betabaculoviruses (granuloviruses (GV) infecting Lepidoptera), Gammabaculoviruses (nucleopolyhedroviruses infecting *Hymenoptera*), and Deltabaculoviruses (nucleopolyhedroviruses infecting *Diptera*).

The major route of infection for baculoviruses is ingestion by the larvae of OBs, contaminating the food. Vertical transmission has been demonstrated for some baculoviruses, but its importance in maintaining the virus populations is still under study (Virto et al., 2013).

Several baculoviruses, NPVs and GVs are used for biological control worldwide.

Researchers in China have isolated 82 virus species from 47 tea pests (Ye et al., 2014). Tea looper NPV virus and tea NPV virus in China have reached the degree of large-scale application. *Cydia pomonella* granulovirus (CpGV) is one of the species that is most used as a bio-pesticide (Berling, 2009).

## Resistance to biocontrol agents

The diversity of the susceptibility of pest populations to biocontrol agents is a first step for the development of resistance. Few studies have described this diversity. For the Cry1F toxin from *Bacillus thuringiensis*, geographical and temporal variability in the susceptibility of *Spodoptera frugiperda* has been shown in populations from Brazil (Farias et al., 2014). Similarly, the variable susceptibility of natural populations to infection with a baculovirus isolate has been described (Briese, 1986; Abot et al., 1996). For instance, Abot et al. (1996) compared the susceptibility of two laboratory colonies to a baculovirus, AgMNPV, and tested the ability of each colony to acquire résistance. These authors showed that the colony sharing the same geographic origin (Brazil) with the virus (where the bio-pesticide is widely used) was able to develop resistance better and faster to the pathogen compared to a colony from the United States.

### Plant extracts

#### Nicotine and neonicotinoids

To our knowledge, resistance mechanisms to nicotine are not addressed in scientific publications. Neonicotinoids, which act onto the same target as nicotine, the nAChRs receptors, are more studied. The results that have been obtained with this chemical family, particularly those concerning the modification of the nAChRs receptors to explain the mechanisms of resistance, may be extrapolated to natural nicotine.

Every modification involving elements of the binding site of nicotinoids and neonicotinoids in nAChRs is a potential source of resistance (Bodereau-Dubois et al., 2012). Modifications of nAChRs can be of two types, either at the pre- or the post-transcriptional level.

Regarding pre-transcriptional modifications, the Y 151 S mutation in the B loop from sub-units α1 and α3 from the *Nilaparvata lugens* leafhopper is correlated with the resistance of this insect to nicotinoids. This mutation induces a decrease in the effects of nicotinoids and of the receptors' sensitivity to these insecticides (Liu et al., 2005, 2006; Yixi et al., 2009). Another type of mutation, this time on the sub-unit Dα1, has also been demonstrated in *Myzus persicae*. The mutation R81T within loop D alters the insecticide binding site, and this amino acid change confers a vertebrate-like character to the insect nAChR receptor and reduces the sensitivity of Myzus persicae to imidacloprid 234 times (Bass et al., 2011).

#### Pyrethrum and pyrethroid

Currently, pyrethrums are far less frequently used than are pyrethroids, which underwent very strong development in the 1970s. The sustained use of pyrethrums and the fact that DDT has the same mode of action have contributed to the development of many cases of resistance. These products act on the same target, and cross-resistances have been demonstrated, especially between pyrethrums and DDT (Lloyd, 1969). DDT itself is subject to cross-resistance with pyrethroids. One can assume that resistance to pyrethroid will also apply to pyrethrins and to pyrethrum. Based on this principle, we will detail the main resistance that is encountered with pyrethroids on insects.

“Knock-down” resistance is the oldest known form of resistance against DDT and pyrethroids. This resistance was first described in a housefly strain (Milani and Travaglino, 1957). The reduction of the sensitivity of the nervous system in the housefly is linked to an allele of the sodium channel gene. This allele is recessive and is called *kdr* for knock-down resistance; it is located on chromosome 3 (Tsukamoto et al., 1965).

A list of mutations in the sodium channel gene conferring resistance to pyrethroids was compiled by Soderlund and Knipple (2003). The emergence of different mutations is correlated with the strong selection pressure, which encourages a reconsideration of the perspectives of sustainability of the use of bio-insecticides made from pyrethrum. The use of these bio-insecticides may be wise in some cases where the target pest would not show pre-established resistance.

#### Neem oil

Very few studies have highlighted the resistance to neem oil for different reasons: the multitude of molecules and potential mode of action of neem extract and the various modes of action that have been identified for purified azadirachtin itself (see paragraph 1.1). However, studies have demonstrated the possible development of resistance (Feng and Isman, 1995) using repeated treatments with either neem oil or purified azadirachtin on peach aphid populations. After 40 generations, these studies observed no resistance for aphids that were treated with neem oil, whereas those that were treated with purified azadirachtin presented a resistance that was 9-fold higher compared to that of non-treated aphids. In extreme conditions, this resistance is equivalent to a low to medium resistance, which could be reached in fields within 4–5 years of exclusive treatments because aphids generally produce a maximum of 12 generations per year. This bio-pesticide seems quite interesting in terms of durability.

### Bacteria and derivates

#### Bacillus thuringiensis

At least 27 species of insects that are able to resist to *Bt* under laboratory conditions have been listed. Many of these resistances concern Lepidoptera (Pardo-Lopez and SoberonBravo, 2013). Thus, 3 species have shown resistance to *Bt*-formulation applied in the field: *Plutella xylostella* (Tabashnik, 1994)*, Trichoplusia ni* (Janmaat and Myers, 2003), and *Plodia interpunctella* (McGaughey, 1985). Many other species are able to develop resistance under *Bt* pressure under laboratory conditions (Table 2).

**Table 2:** **Cases of resistance of various insect species to *Bacillus thuringiensis* under field or laboratory conditions**.

**Insect species**	**Product**	**Lab/Fields**	**References**
*Heliothis virescens*	*Bacillus thuringiensis var. kurstaki*	Only in laboratory	Macintosh et al., 1991
*Leptinotarsa decemlineata*	*Bacillus thuringiensis*	Only in laboratory	Whalon et al., 1993
*Ostrinia nubilalis*	*Bacillus thuringiensis var. kurstaki*	Only in laboratory	Huang et al., 1997
*Plodia interpunctella*	*Bacillus thuringiensis var. kurstaki* Et autres *Bt*	Fields	McGaughey, 1985; McGaughey and Beeman, 1988; McGaughey and Johnson, 1992
*Plutella xylostella*	*Bacillus thuringiensis var. kurstaki*	Fields	Tabashnik, 1994; Wang et al., 2007
*Pseudoplusia includens*	*Bacillus thuringiensis* var. *kurstaki*	Only in laboratory	Mascarenhas et al., 1998
*Spodoptera exigua*	*Bacillus thuringiensis*	Only in laboratory	Moar et al., 1995
*Spodoptera littoralis*	*Bacillus thuringiensis*	Only in laboratory	Mullercohn et al., 1996
*Trichoplusia ni*	*Bacillus thuringiensis* var. *kurstaki*	Greenhouse	Janmaat and Myers, 2003

*The resistance of mosquitoes and the resistance of insects to Bt GMO crops were not considered*.

Resistance to *Bt* can occur at any step of the infection cycle. This literature presents some examples of mechanisms that have been discovered at different levels: (i) crystal degradation by proteases, (ii) the absence of activation of the crystal by N-aminopeptidases, (iii) the alteration of the protein-binding membrane or (iv) the formation and insertion of the membrane pore. The most common phenotype of resistance to *Bt* within Lepidoptera is called “modus 1” and is characterized by a high level of resistance (500 times higher compared to a sensitive strain) for at least one toxin of the Cry1A family. Modus 1 has recessive transmission and a very low or no cross resistance with other toxin of the Cry1C family. Modus 1 resistance has been reported for at least one *Plutella xylostella*, *Heliothis virescens*, and *Plodia interpunctella* strain. Although certainly common, modus 1 is not the only phenotype of *Bt* resistance (Tabashnik et al., 1998). Scientific studies of these mechanisms are mostly specific to a Cry acting in one insect species. The following mechanism analysis is not exhaustive.

##### Alterations implying primary and/or secondary receptors

The most represented resistance mechanisms consist of the reduction of the Cry toxin-epithelial cell membrane link in different insect species, including mutations or loss of receptors, such as cadherin, alkaline phosphatases (ALP) or aminopeptidase N (APN) (Bravo et al., 2011).

Ferre et al. (1991) demonstrated by comparing a resistant strain of *Plutella xylostella* that was collected from a field to a sensitive laboratory strain that the resistance difference came from a lower affinity of the Cry1A toxin with the membrane receptors of the epithelial cells. Gene mutations decreasing or destroying the expression of cadherins are the origin of Cry1A resistance of *Heliothis virescens* and *Helicoverpa armigera*. Nevertheless, the molecular nature of these mutations is not clearly known (Ferre and Van Rie, 2002; Heckel, 2012). Many genes that are responsible for *Bt* resistance by cadherin alterations have been identified. Among the 12 identified alleles that are responsible for Cry1A resistance related to cadherins, one concerns *H. virescens*, three *P. gossypiella*, and eight *H. armigera* (Gahan et al., 2001; Morin et al., 2003; Xu et al., 2005; Zhao et al., 2010). Similarly, Liu et al. (2000) showed that the mechanisms that are involved in resistance differ between Cry natures. Indeed, in *P. xylostellales*, the resistance mechanisms to Cry1C were different from those that are involved in Cry1A resistance and were not sufficient to induce a resistant phenotype.

Regarding the APN receptors, Herrero et al. (2005) revealed the absence of one of them in a *Spodoptera exigua* strain that is resistant to the Cry1Ca toxin. A northern blot analysis of the 4 N-aminopeptidase expression levels revealed that the gene coding for APN1 was not expressed, while the 3 others had an equivalent expression level in either resistant or sensitive populations. Similarly, Tiewsiri and Wang (2011) showed that the resistance of *Trichoplusia ni* to the Cry1Ac toxin could be explained by a reduced number of secondary protein receptors, APN1, and the fitness loss would be compensated by APN6, which is not involved in toxin binding. These alterations would not be the result of APN1 and APN6 genes mutations but more likely putative mechanisms of trans-regulation at the transcription level of these genes. In a resistant laboratory strain of *H. armigera*, a deletion in the HaAPN1 receptor gene is involved in resistance to Cry1Ac but not in another resistant strain from the field (Zhang et al., 2009). More recently, Khajuria et al. (2011), after the analysis of 10 genes that were linked to aminopeptidases, observed no differences in the expression between resistant and susceptible strains of an *O. nubilalis* population to Cry1Ab. However, a change in two amino acid residues of a P-aminopeptidase (APP) coming from two resistant strains could demonstrate the implication of the APP mutation in the resistance.

*Bt* resistance does not systematically implicate mutations in genes coding for proteins in the membrane of the epithelial cells because susceptible and resistant populations of *S. exigua* have the same expression level of those genes. Soluble APN in the intestinal lumen acts as a competitive inhibitor of the membrane form, preventing the fixation of Cry. These soluble ANPs are present even without infection by *Bt*, which may implicate the immune system. This resistance could be the result of the constitutive activation of APN genes (Hernandez-Martinez et al., 2010).

##### Alterations involving an ABC transporter

A second independent mechanism has been identified, implying an ATP-binding cassette transporter (ABC transporter). The discovery of this mechanism resulted in the identification of a resistance allele to Cry1Ac in an *H. virescens* population, mutated in a gene encoding the ABC molecular transporter. A simple mutation of domain 12 of cadherin could induce resistance but alone will not explain the loss of a link between the toxin and the membrane (Gahan et al., 2010). The opening of the membrane by the ABC transporter would promote the insertion of the preformed pores. The exact action of this ABC transporter is not totally understood, even if its implication has been demonstrated in Bt resistance (Soberon et al., 2012). Subsequently, a loss of ABC2 protein expression, which is genetically linked to resistance to the Cry1Ac toxin, was discovered in Plutella xylostella and T. ni (Baxter et al., 2011).

##### Protease implication

**Figure 1:**
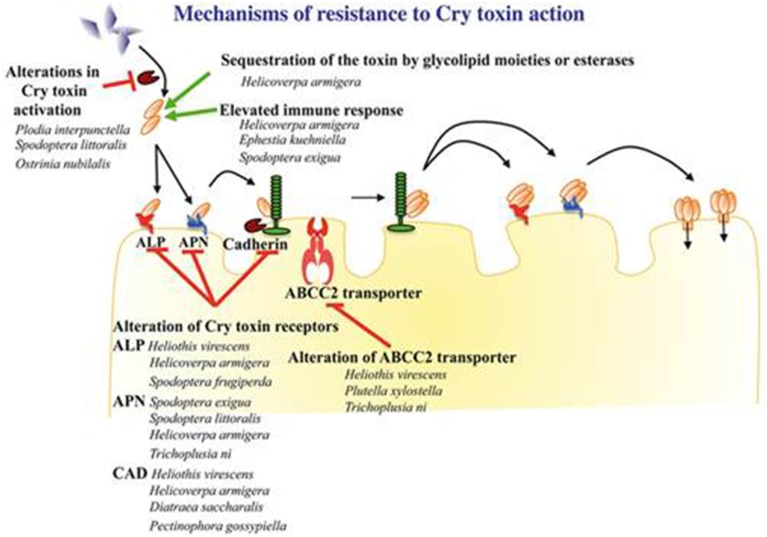
**Schematic representation of the different mechanisms of resistance to the 3d-Cry toxin as described in Lepidoptera insects (Reproduced with permission from Pardo-Lopez and SoberonBravo, 2013)**.

Digestive proteases play an important role in insect infection by the activation of toxins. The observation of a reduced Cry1A protoxin hydrolyzation level in Plodia interpunctella populations that were resistant to Bt (Oppert et al., 1997) indicates a possible reduction in the proteolytic activity, resulting in the less efficient trypsin digestion of the protoxin. Similar mechanisms have been demonstrated in Ostrinia nubilalis (Li et al., 2004) and in Bt-resistant *H. virescens* populations, where a protease alteration binding to Cry1Ac could play a role in the gain of resistance (Karumbaiah et al., 2007). This alteration is based on a lack of binding to one of the three recognition sites of the enzyme, inhibiting their activity. The implication of this mechanism into the resistance process is poorly known, although Herrero et al. (2001) revealed that, in *P. interpunctella*, proteinase alteration accounts for 90% of the resistance. Figure 1 summarizes the mechanisms that are involved in resistance to Cry1Ac for several insects.

#### Spinosyns

The first occurrence of resistance to spinosyns has been highlighted in the laboratory with *Heliothis virescens* (tobacco budworm) and *Musca domestica* (house fly) populations. Other resistances have been identified, for example in *Drosophila melanogaster* (fruit fly), *Liriomyza trifolii* (leaf miner), *Helicoverpa armigera* (bollworm cotton), *Plutella xylostella* (diamondback moth), *Frankliniella occidentalis* (western flower Thrips), and *Spodoptera exigua* (beet army worm). These pests show wide levels of resistance in the laboratory (Sparks et al., 2012), and 6 species (two dipterans, three Lepidoptera and one Thysanoptera) showed resistance to spinosyns in the field (Table 3). The resistance levels of *Plutella xylostella* and *Frankliniella occidentalis* are extremely high compared to those the other insects (Table 3).

**Table 3:** **Resistance to spinosyns and the involved mechanisms (Sparks et al., 2012)**.

**Insect species**	**Insecticides**	**Resistance ratio**	**Mechanism involved**
*Bactrocera oleae*	Spinosad	10–13	–
*Liriomyza trifolii*	Spinosad	>118^a^–1192^b^	–
*Helicoverpa armigera*	Spinosad	4–9,5	Cytochrom P450
*Plutella xylostella*	Spinosad	>20000	Target mutation
*Spodoptera exigua*	Spinosad	33–158	Cytochrom P450
*Frankliniella occidentalis*	Spinosad	13500	Target mutation
*Drosophila melanogaster*	21-butenyl A	265	Target mutation
	Spinosad	1181	
	Spinetoram	176	

a *In the 4 months prior to collection, CA-1 strain had multiple applications of cyromazine, abamectin and spinosad*.

b *Just prior to collection, CA-2 strain had multiple applications of abamectin and spinosad; no cyromazine*.

In most cases, resistance comes from a strong selection pressure due to an intensive use of the spinosyns (Sayyed et al., 2004). For example, *Plutella xylostella* became resistant to spinosyns after only two years of use in Hawaii. This product had been released on the Hawaiian market in 1998 to control populations of *P. xylostella*, which is resistant to chemical insecticides. In a very short time, spinosad became the most employed control method against this pest. Between 2000 and 2002, a bioassay on *P. xylostella* populations showed resistance to spinosyns throughout the entire island of Hawaii.

In a resistance management context, spinosad was withdrawn from the Hawaiian market and was temporarily replaced by other insecticides before being reintroduced two years later. Following its reintroduction, a re-emergence of resistant individuals occurred in some areas of the island (Zhao et al., 2002). The development of resistance was probably due to monocultures of Cruciferae, on which repeated treatments of spinosad were performed in an isolated population of *P. xylostella*.

A cross-resistance event was highlighted between different spinosyns. For example, populations of *Drosophila melanogaster* are resistant to both spinosad and 2-butenyl (analogs of spinosyns A isolated from *S. pogona*). Mota-Sanchez et al. (2006) identified a possible cross-resistance between imidacloprid and spinosad in populations of *Leptinotarsa decemlineata* (Colorado), a major pest of potatoes in Latin America and Europe. Sayyed et al. (2008) reported cross-resistance to indoxacarb in *Spodoptera litura*. Dunley et al. (2006) showed cross-resistance in populations of the leafrollers *Choristoneura rosaceana* and *Pandemis pyrusana* in Washington apples.

Spinosyn resistance mechanisms are not fully understood; however, two mechanisms seem to be involved. A very large part of the resistance can be attributed to a molecular target change. This change has been demonstrated in several pests (Sparks et al., 2012). For example, a modification in the Dα6 sub-unit from the nAchR receptor of *Drosophila melanogaster* creates very strong resistance, suggesting that this sub-unit is the anchor of the spinosad (Perry et al., 2007). However, the resistance of *Plutella xylostella* is due to alternative splicing in the sub-unit α, leading to the premature formation of a stop codon inactivating the nAchR (Baxter et al., 2010; Rinkevich et al., 2010).

### Fungus

The mechanisms that are involved in resistance to fungi are usually defense mechanisms, mainly protease inhibitors, such as serine protein inhibitor (SPI), trypsin inhibitors (TIs), chymotrypsin inhibitors (CIs), elastase inhibitors and subtilisin inhibitors. In *Bombyx mori*, 14 SPIs are over-expressed during a pathogenic infection, such as SPI 11 during infection with *Beauveria bassiana* (Zhao et al., 2012). The inhibiting action of SPI 38 on many virulence factors, such as CDEP-1 of *B. bassiana*, K protease of *E. album*, subtilisin A of *B. licheniformis*, and AmP of *A. melleus*, was also attested (Li et al., 2012a).

Another type of gene involved in these resistance phenomena is an AFP (antifungal protein). This gene was found in *Sarcophaga peregrina*, and its action was described in the yeast *Candida albicans*. AFP represses fungal growth, and a synergistic action with a sarcotoxin IA, which is present in the hemolymph of insects during infection, has been demonstrated. From binding experiments, it appears that AFP binds to nucleic acids and that this binding is blocked in salt solution, but the protein preserves its antifungal activity (Iijima et al., 1993).

### Viruses

Lepidoptera larvae become increasingly resistant to NPV infections as they age. Briese (1986) summarized the variation for 20 insect species. This author reported resistance ratios reaching to 350,000-fold for *Pieris brassicae granulovirus* but only 10-fold for *Cydia pomonella granulovirus* on their respective hosts, justifying the importance of early treatments when using baculoviruses for pest control. It has been proposed that this resistance may reflect the increase in the weight of the host; although no dose increase is required when the virus is injected (Engelhard and Volkman, 1995).

Variations in the susceptibility to a given virus isolate between insect populations from different geographic origins have also been observed (Briese, 1986). Even within a single population, the presence of individuals with a reduced susceptibility was noted for the codling moth (Sheppard and Stairs, 1977). These differences have been related to the specific genetic background of the populations. In the potato tuber moth, *Phthorimaea operculella*; the reduction in the susceptibility to a Phthorimaea operculella granulovirus (PhopGV) isolate was due to a single dominant autosomal gene. This reduction resulted in a 15-fold resistance ratio (Briese, 1986). In the armyworm *Spodoptera frugiperda*, a fivefold difference in the susceptibility of two laboratory colonies to three different baculoviruses has been attributed to a single or few autosomal genes (Reichelderfer and Benton, 1974). In contrast, in the codling moth, this susceptibility appears to be under multigenic control (Berling et al., 2013). Maintaining the differences in susceptibility between insect populations does not seem to rely on the presence of the virus. David and Gardiner (David and Gardiner, 1965) reported that two colonies of *Pieris brassicae* showing differences in susceptibility to two isolates of the Pieris brassicae granulovirus (PbGV) maintained the differences after 36 generations in the laboratory in the absence of virus contact.

Under laboratory conditions, Abot et al. (1996) reported that populations of the velvet worm *Anticarsia gemmatalis* collected from Brazil and the USA could develop resistance when exposed to the LD-79 isolate of AgMNPV. Resistance began to develop after 4-5 generations and reached a plateau after 15 generations. The final level of resistance (up to a 17,000-fold increase) depends on the population origin, suggesting that the original genetic variability of the host population is an important factor. However, in Brazilian fields, the use of AgMNPV to control the velvet worm represents approximately one million hectares, but no increase in the dose is required to control the insect.

There are different hypotheses regarding the mechanisms that are involved in resistance. Fuxa and Richter (1990) showed that the mortality of the sensitive and resistant larvae of *Spodoptera frugiperda* did not differ when the virus is injected into the hemocele and concluded that resistance is associated with midgut cell penetration. Detailed studies have revealed increased difficulty for the virus to transfer the infection from the midgut cells to the rest of the larvae. The first mechanism relies on the capacity of the host to renew midgut cells by sloughing, permitting the elimination of infected cells during larval development. During molting from the 4th to 5th instars in *T. ni*, all infected of the midgut cells were eliminated (Engelhard and Volkman, 1995). There have been no observations indicating whether during molting from each instar to the next, all of the midgut cells are renewed. The second mechanism that has been proposed is the disruption of the peritrophic membrane (PM), which would protect the infection of midgut cells. The presence of stilbene-derived optical brighteners reduces or abolishes this developmental resistance (Murillo et al., 2003). Optical brighteners interfere with the synthesis of chitin, one of the components of the PM (Wang and Granados, 2001). In addition, an analysis of the structure of the PM, which presented differences between AgMNPV-susceptible and -resistant laboratory colonies of *A. gemmatalis*, suggested its involvement in resistance (Levy et al., 2007). However, optical brighteners also seem to prevent midgut cell sloughing (Washburn et al., 1998). Grove and Hoover (2007) observed a variation in the susceptibility within the same larval instar in *Lymantria dispar* that is not limited to the midgut but is systemic. These authors suggest that the observed effect is the result of anti-viral defenses that are hormonally controlled.

From 2003, failure of codling moth control on orchards that were treated by CpGV-M was detected in Germany (Fritsch et al., 2005) and then in other countries in Europe (Sauphanor et al., 2006; Schmitt et al., 2013). The resistance in field populations reached 1000-fold, but this ratio in laboratory colonies of pure resistant genotypes, such as *C. pomonella* R_GV_, is as high as 60,000-fold (Berling et al., 2009).

Asser-Kaiser et al. (2010) showed that the resistance of the codling moth to CpGV is not conferred by a blockage at the midgut level either by the modification of the PM or by the modification of brush border cell receptors. The resistance is generalized to all cells in the insect; the virus enters, but replication is blocked at an early phase (Asser-Kaiser et al., 2010). A virus-cell incompatibility is suggested. Interestingly, this resistance is restricted specifically to the CpGV-M genotype; other isolates, such as CpGV-I12 (Eberle et al., 2008) or CpGV-R5 (Berling et al., 2009), are not inhibited. The mechanism underlying this resistance is not known, even though *pe38*, the virus gene that is involved in the difference between isolates able to replicate (ex CpGV-R5) or not (CpGV-M) in resistant larvae, has been identified (Gebhardt et al., 2014).

### Cross resistances and detoxification systems

Recombination tests expressing Cry1Ab showed that a cross-resistance to CpGV and *Bt* is not likely (Asser-Kaiser, 2009). There are few recorded cases of cross-resistance to organic insecticides. Sayyed et al. (2004) showed the existence of a population of *Plutella xylostella* “CH1” from the field that is resistant to several insecticides (Multidrug resistance) that do not have the same mode of action or the same target. This resistance relates to spinosad and *Bt* Cry1Ac.

However, there are many cases of cross-resistance between spinosad and insecticides belonging to the chemical class of neonicotinoids. Because they have the same molecular target, a change in nAChR may result in a lower affinity to several products (Puinean et al., 2013; Bao et al., 2014). This consideration is also true for both pairs natural pyrethrum / pyrethroid (Soderlund and Knipple, 2003) and nicotinoid/neonicotinoids (Bodereau-Dubois et al., 2012).

Similarly, cell detoxification mechanisms selected from chemical insecticides can also enable the degradation and expression of organic insecticides. For example, Dunley et al. (2006) reported that populations of *Choristoneura rosaceana* and *Pandemis pyrusana* are resistant to azinphos-methyl, tebufenozide, spinosad, and methoxyfenozide. This mechanism of resistance is different from the molecular target modifications previously described; it consists of the sequestration, elimination or metabolization of the xenobiotic products. These processes can involve enzymes with P450 cytochromes, which introduce an oxygen atom into their substrates; esterases (or hydrolases), which cleave esters and amides, thus increasing the polarity of the metabolites; or glutathione-S-transferase, which catalyzes the conjugation of molecules with an electrophilic center with the thiol group of the glutathione.

P450 cytochromes mono-oxygenases are involved in the insect metabolism of juvenile hormones and ecdysones, in the synthesis of pheromones, and in the protection against plant toxic substances. The P450 cytochrome enzymatic systems are often compared to the components of the immune systems because of their implication in defense reactions. This comparison is valid because the synthesis of many P450 cytochrome enzymes is induced in the presence of the toxic substances that they metabolize. The role of the over-expression of the gene coding for the enzyme CYP6G1 (an oxidase) in the resistance of *Drosophila melanogaster* to nicotine (Li et al., 2012b) has been studied. Housefly resistance to spinosad is also partly due to P450 cytochrome mono-oxygenases. Markussen and Kristensen (2012) report that this resistance is female-linked, with a negative cross with neonicotinoids in one strain, and the alteration of cytochrome P450 gene expression. In both cases, an addition of an activity inhibitor of the mono-oxygenases, such as piperonyl butoxide (PBO), permits the recovery of some of the product effectiveness. This molecule is sometimes called a “synergist” of the insecticide action in some cases of resistance to various insecticides. Many commercial products based on pyrethrum contain PBO to increase its effectiveness. Resistance to spinosad is attributed to detoxification by p450 in the following pests: *Musca domestica* (Hojland et al., 2014), *Thrips palmi* (Bao et al., 2014), *Cydia pomonella* (Reyes and Sauphanor, 2008), *Plutella xylostella* (Pu et al., 2010), *Bemisia tabaci* (Wang et al., 2009a), and *Helicoverpa armigera* (Wang et al., 2009b).

However, the increase in GST and esterase activities under conditions of stress as induced by the presence of nickel at sublethal doses does not necessarily increase the resistance of *Galleria mellonella* to *B. bassiana*. In contrast, an increased susceptibility is noted by Dubovskiy et al. (2011). Enzyme-induced defenses seem to be specific to the stress to which the insect is subjected. One case of resistance to *Bacillus thuringiensis* subsp *israeliensis* (*Bti*) in *Aedes rusticus* larvae (Diptera: Culicidae) was reported and presumably attributed at a detoxification mechanism by GST (Boyer et al., 2012). In addition, Gunning et al. (2005) reported that the sequestration of the *Bt* toxin Cry1Ac in a tolerant *H. armigera* strain is due to toxin binding to esterases. There is no case of resistance to bio-insecticides, implying insect behavioral changes or the direct excretion or penetration reduction of a bio-insecticide, whereas there are well-documented mechanisms with chemical insecticides.

## Sustainability of biocontrol agent efficacy

The ability of pests to become resistant to chemical pesticides has been widely studied. This topic has been the subject of various scientific articles and books (Georghiou and Taylor, 1977; Roush and Tabashnik, 1990). Here, we will summarize these findings and include what differentiates bio-pesticides from their chemical counterparts.

### Resistance risks factors

#### Genetic factors in the pest

The various factors (here underline) that could influence resistance emergence were initially defined for chemical insecticides, but most of them also apply to biocontrol agents. The first factors are related to genetics of the pest. The presence of mutations conferring a pre-existing resistance includes insects showing resistance to chemical products that can also be resistant to innovative products, which could be the case for nonspecific resistance mechanisms as well as specific mechanisms if the molecular target is the same (example: pyrethrum with synthetic pyrethroid or DDT). The higher is the number of mutations that are required for resistance, the lower is the risk of occurrence of resistance. The dominance level of the resistance allele has also an impact on both the maintenance and dispersion speed of resistance (Comins, 1977; Taylor and Georghiou, 1979; Curtis et al., 1987). This level will determine if heterozygous individuals are killed by the pesticide. This factor is particularly influential when sensitive pest are introduced to a resistant population (Tabashnik and Croft, 1982). Studies have demonstrated that it is easier to handle dominant monogenic resistances than recessive polygenic resistances (Hoy, 1985). The identity of the chromosome carrying the resistant allele also influences the mode of transmission of the resistance. We illustrate this statement with two examples of resistance to the BCA with different transmission modes.

The heredity of CpGV-M resistance was first described as monogenic and sex-linked determinism (Asser-Kaiser et al., 2007; Berling et al., 2007). Asser-Kaiser et al. (2010) showed that the level of resistance was not similar between heterozygous and homozygous males, suggesting a gene dosage effect. The very strong efficiency of the resistance and this effective mode of transmission were brought to rapid selection under constant pressure of the virus (Berling, 2009). Underlying this major resistance, the genetic background also plays a role (Berling et al., 2013).

Research on the mode of transmission of *Bt* resistance has revealed the lack of a unique mechanism. Kaur and Dilawari (2011), under laboratory conditions, showed that the resistance of *Helicoverpa armigera* to Cry1Ac was autosomal, partially recessive and polygenic. Similar conclusions were obtained for populations of *Plutella xylostella* (Sayyed et al., 2000), *Heliothis virescens*, and *Pectinophora gossypiella* (Kaur and Dilawari, 2011). However, studies with *P. xylostella*, revealed that the sex of the parent carrying the resistance to Cry1Ab was important to the survival of the F1 generation (Martinez-Ramirez et al., 1995). A similar observation was reported for a population of *S. littoralis* with Cry1C (Chaufaux et al., 1997). The genetics of resistance appear sometimes variable between populations of the same species. *Plutella xylostella* is a good illustration. A population coming from Hawaii carries a trait of resistance to Cry1Ac that is partially dominant, while a population from South Carolina carries one trait that is partially recessive. Other studies have reported that this resistance was completely recessive. Are we in the presence of a complex set of alleles showing different dominances? (Kaur and Dilawari, 2011). The picture is even more complex, as the apparent dominance seems to be related to the dose ingested. Sayyed et al. (2000) noted that the amount of toxin that was ingested affected the dominance of the resistant allele to Cry1Ac in *P. xylostella*. In the presence of low concentrations, resistance is almost completely dominant, whereas for higher concentrations, resistance is recessive. Increasing the dose of toxin can be efficient only if the resistance is not fully dominant. In contrast, if the resistance is dominant, high doses of *Bt* lead to a rapid increase in the resistance frequency in the insect population.

The frequency of resistant individuals in the case of monogenic resistance increases proportionally to the logarithm of the initial frequency of the resistance allele (Tabashnik and Croft, 1985). For example, Bourguet et al. (2003) and his team suggested that the initial frequency of a resistance allele to *Bt* in *Ostrinia nubilalis* from the northern USA and France is probably rare enough to delay resistance. Last but not least, genetic factors affect the incidence of resistance on insect fitness in the absence of selection pressure. This factor is one of the most determining factors for the propensity and the stability of resistance in the field (Georghiou, 1983). Resistant individuals frequently have lower fitness in the absence of pesticide. The pleiotropic cost includes features such as the rate of development, survival rate, fecundity, fertility, sex ratio reproductive and dispersive capacity. Resistance to *Bt* decreased in the absence of selection in a large number of laboratory strains (Janmaat and Myers, 2003). Under these conditions, resistance at the population level is reversible, and a susceptible population can quickly be resettled. Gassmann et al. (2009) reviewed 77 studies, including 18 species, and a biological cost of resistance to *Bt* was detected in nearly 60% of the cases. These authors report that in most cases, this cost affects the recessive resistance and that the non-recessive cases were more strongly selected against resistance. The changes in the cadherin genes, the proteases and the aminopeptidases constituted a biological cost of resistance that could have serious consequences on the food performances of these insects.

Concerning baculoviruses, the resistance that was observed in *A. gemmatalis* after selection against AgMNPV reverted to the original levels after a few generations without virus treatment, suggesting a selective cost for maintaining the resistance (Fuxa and Richter, 1989, 1998). However, a different figure is observed for *C. pomonella* resistance to CpGV-M. The resistance remained stable for 30 generations without contact with the virus, and only a 10-fold reduction was obtained after 60 generations (Undorf-Spahn et al., 2012), suggesting that, at least under laboratory conditions, no (or very reduced) fitness cost is associated with maintaining this resistance.

#### Biological and ecological factors in the pest

The number of generations per year is directly proportional to the resistance evolution rate (Tabashnik and Croft, 1985). The pest might develop a resistance faster because the selection process is accelerated when the number of generation per year is high. Therefore, insects with shorter cycles are more likely to develop resistance. Similar to the number of generations per year, the fecundity accelerates the rate of multiplication of an individual and consequently the chances of transmitting resistance trait. The more polyphagous is the pest, the wider is its potential area of spreading, and the higher is the propagation of the character of resistance. However, if agricultural practices differ between cultures (the use of different products for instance), the possible benefits for the resistance that is provided by that factor are canceled. The reservoir of susceptible genotypes is a crucial factor for the evolution of resistance. The immigration of susceptible individuals to treated areas can reduce the development of resistance by increasing the frequency of susceptible alleles (Comins, 1977; Georghiou and Taylor, 1977; Taylor and Georghiou, 1979; Tabashnik and Croft, 1982; Curtis et al., 1987). These models also demonstrate that the rates of resistance decrease as the ratio of sensitive migrants in the treated area increase. Models have been developed to optimize these refuges in a context of resistance management to *Bt*-corn (Tyutyunov et al., 2008).

In contrast, the emigration of resistant individuals from a treated to an untreated area increases the rate of development of resistance in that area (Comins, 1977; Taylor et al., 1983). Therefore, pest mobility is the last factor considered in this section.

#### Factors related to the biocontrol agent

The novelty of the biocontrol agent (BCA) and the originality of this mode of action are the first factors. If the molecular target of the bio-pesticide is already under selection pressure by other pesticides, its pressure will increase the propensity to develop resistance. The low persistence of the products in the environment will lead the grower to treat several times, thus selecting resistant individuals faster. Inversely, more persistent products will allow for the application of less insecticide. A high persistence could submit pests to a continuous selection pressure and lead to the selection of resistant individuals. It is necessary to find the right trade-off between frequency of application and persistence. The detoxification capacity (in the case of extracts) states that the pre-adaptation hypothesis postulates that pests are pre-adapted to detoxify the (bio) pesticides because they have already developed systems that allow them to detoxify compounds generated by plant defense mechanisms, such as alkaloids, terpenoids, phytosteroids, and other allele-chemicals. Such studies have confirmed these hypotheses on phytophagous species, although there are some exceptions (Rosenthal and Janzen, 1979; Berenbaum, 1985; Berenbaum and Neal, 1987). The risk of cross-resistance is estimated from the original baseline susceptibility (see general introduction). Finally, the risk of resistance will be much higher for a “uni-site” than for a “multi-site” insecticide or bio-insecticide. For a multi-site bio-insecticide, the diversity of targets will significantly reduce the risk of resistance. However, cases of resistance to *Bt* (a famous multi-sites insecticide) have already been reported. It would be interesting to include the use of bio-pesticides in a more integrative control method that includes a set of practices to reduce the appearance of resistance: integrated control.

#### Operational or agronomic factors

The number of host plants that are treated is directly linked to the number of pest host plants, as well as the territory fragmentation. The less fragmented is the territory, the easier and faster the resistance will appear. The number of applications / number of treated generations is directly linked to the resistance allele frequency. The speed of resistance emergence is therefore proportional to this factor. The existence of generations free from selection pressure can greatly reduce the rate of emergence of resistance. Rotations promote breaks in the proliferation of a specific pest and thus in its resistance. Monoculture is a detrimental factor in maintaining the long-term efficiency of a product. The applied dose could also be a risk factor. When a dose selects the resistant individuals at the expense of the susceptible ones, it could favor an increase in the frequency of resistant individuals. The landscape structure will influence the dissemination of pests and more precisely of resistant pests. Compared to traditional farming systems, organic farming uses a larger number of applications, but crop rotations are almost systematic. As organic farming remains marginal, treated plots are disseminated in the landscape, favoring resistant and susceptible population mixtures. In total, operational and agronomic factors should not cause a greater risk of resistance emergence to the BCA compared to chemical insecticides.

In summary, the three most influential factors in the estimation of the risk of resistance are the reproductive factor (number of generations per year + fertility), the dispersive capacity, and the fitness cost. A few of these factors are alterable by human action: the operational/agronomic factors and the factors that are related to the BCA. Among these factors, the number of treatments and the proportion of the treated population (number of treated host plants + reservoirs of susceptible genotype) are the two main parameters that can influence the apparition of resistance (Tabashnik and Croft, 1982; Regnault-Roger et al., 2002). Most models anticipate the reduction of the number of treatments. This approach has been called management by “moderation” or the “decreased pesticides use” strategy. However, this strategy is not always possible in the field. Prior to their marketing authorization, it would be appropriate to have an analysis of risk factors for organic control products to assess their potential sustainability.

The power of living organisms, such as fungi, viruses or bacteria, is their potential to co-evolve with the pest if they are allowed to. Because of the complexity of the mechanism of host-pathogen interactions, it is much more difficult for the pest to overcome the treatment. In return, the pathogen does not completely eliminate its host. Purified extracts of plants or bacteria that have a single target in the insect host have properties that are closer to those of chemical insecticides; they cannot adapt. Therefore, the risk of resistance is higher. Finally, the long co-evolution history of host and pathogens is responsible for the weaker efficiency compared to that of chemical molecules, which in return is a guarantee of durability.

### Management strategies

Risk management reduces risk by (i) reducing the potential impact of the hazard, (ii) reducing the probability of the hazard occurring and (iii) reducing the exposure.

Based largely on theoretical work, several tactics have been proposed by Georghiou (Georghiou, 1983) for use in integrated resistance management strategies. These tactics fall into three categories:

Management by moderation aims to reduce selection for resistance by preserving susceptible insects in the population through the use of low-application doses, less frequent applications, short-lived residues, or the creation of untreated refuges. Lowering application rates will only help when managing resistance under very specific circumstances and if not will drastically lower the efficacy of the control.

Management by saturation aims to overcome resistance using doses that are sufficiently high to kill even resistant insects and/or using compounds toward which resistance is weaker. In some cases, “high-dose” strategies have been proposed to prevent a first emergence of resistance by the elimination of heterozygous resistant individuals (Georghiou, 1983; Roush and Tabashnik, 1990). However, these strategies probably apply only to cases where the exposure of the insect to insecticides is relatively uniform and can be precisely controlled.

Management by multiple attacks involves the use of two or more unrelated insecticides to reduce the selection of resistance to each of them. The compounds can be applied simultaneously as mixtures that are alternated over time or applied in more complex spatial patterns known as mosaics.

Are these principles applicable to biocontrol agents? The availability of products in the market is a requisite for their use with a multiple attack strategy. The use of product mixtures is often prohibited for bio-insecticides because of the nature of these products, which are more fragile and technical than are chemical molecules. To implement an efficient strategy against bio-pesticide-resistant insects, new products are needed, together with a wide knowledge of their mechanisms of action. Research is necessary to allow sustainable control in modern agriculture.

Moreover, to limit the emergence of resistance to bio-insecticides, they must be used according to the defined policies. The use of chemical insecticides should be a last resort. It is important to develop a large variety of methods to reduce pest damage and put diverse selection pressures in place.

In addition to the implementation of these strategies, it is important to monitor the effectiveness of the treatments that are used to detect the emergence of resistances as soon as possible. In the event of ineffective treatment, questioning is necessary. If resistance is the cause, the pest control strategy should be rapidly redrafted to limit the selection of resistant individuals.

## Conclusion

Resistance to insecticides is a major problem for agriculture and for the control of disease vectors. Cases of resistance have been reported since the 1950s, although they have been considered in recent years. Bio-insecticides include a wide variety of compounds and organisms to ensure plant protection. One would expect that the diversification of the molecular and biochemical targets in pests could limit emergence of resistance (Regnault-Roger et al., 2002). The use of a limited number of targets in a systematic and repeated manner, however, generates cases of resistance. This figure is more striking when the bio-insecticide is a purified product (a single molecule, such as a specific *Bt* toxin). Under these conditions, there is no fundamental difference between a synthetic molecule and a bio-insecticide. We can extrapolate the figure for the use of a single virus genotype. When the full complexity of the agent is retained, as when using complete *Bt* spores and crystals, crude neem extracts, or genotypically diverse virus populations, no resistance development has been observed.

This bibliographical study shows that a broad proportion of resistance cases are due to a specific mutation or post-transcriptional modifications in the molecular targets of the biocontrol agent (Lloyd, 1969; Perry et al., 2007; Baxter et al., 2010; Rinkevich et al., 2010; Bravo et al., 2011; Bodereau-Dubois et al., 2012). Cases of the development of aspecific resistances are relatively rare compared to chemical insecticides, even if differences in susceptibility are found between geographically different insect populations. This observation, coupled with the specific mode of action of each biocontrol agent, leaves little probability of cross-resistant development.

The fitness cost that is associated with resistance is very variable. The genetics underlying the transmission are also variable, multigenic or monogenic, recessive or dominant.

These facts prove that resistances to bio-pesticide molecules are likely to arise in the future and will be various and diverse but are less likely when increasing the diversity of the biocontrol agents.

Cases of resistances to living organisms, such as entomopathogenic fungi, provide a good outline of the complexity of co-evolving systems.

Following the comparison, we realize that most of the durable varietal resistances are those that are multigenic. The long co-evolution of a plant and its pest or of an insect pest and its biocontrol agent will support the development of complex strategies, which will not be commonly avoidable by a single mutation. In contrast to our general thinking some years ago, resistance to a virus genotype in the field has occurred. In the case of codling moth resistance to the granulosis virus (CpGV-M), a very strong resistance is mainly conferred by a dominant allele, making this product completely ineffective in resistant populations. Other genotypes that are present in viral natural populations control these resistant insects. It has been recently suggested that the CpGV-M genotype corresponds to the recent evolution of CpGV (Eberle et al., 2009) which would suggest that this genotype has a selective advantage (for example, a higher progeny yield in the larvae). The presence of such an advantage would be frequency-dependent in a partially resistant host population but represents a clear advantage if the host is fully susceptible. Soon after the first isolation of CpGV-M, Sheppard and Stairs (Sheppard and Stairs, 1977) noted the variable response of hosts, suggesting that codling moth populations already comprised some resistant individuals. Although the genetic variability of natural populations of baculoviruses has been detected since the earlier works on Restriction Enzyme Length Polymorphism analysis of baculovirus (mainly NPV) genomes, its relevance in terms of biological control is quite recent. The registration process, which was mainly inspired on chemicals, was developed for the most homogeneous possible active ingredient, which is a pure genotype. It was only after the REBECCA European project and the emergence of resistances that the importance of the diversity of a virus population began to be considered.

The management of resistance to bio-insecticides is essential for a long-term vision of biocontrol. Consequently, these new resistances must be monitored, studied and managed. Bio-insecticides, which are considered universal resistance-proof methods, appear to present risks. The key point is the management of the selection pressure by the following: (i) The diversification of the control methods on fragmented areas will reduce the pressure of selection and especially the spread of resistant individuals. The complexity of action for biological control agents is maintained, as the acquisition of resistance requires more significant changes in these cases, and resistance is therefore less likely. (ii) Allowing continuous co-evolution between the host pest and its pathogen, resulting in the compensation of specific host mutations. (iii) Maintaining a careful and detailed monitoring of the development of resistance to react quickly to the first confirmed cases.

This study highlights the necessity for the proper management of these new products to avoid repeating previous mistakes. In addition, it appears that pest control strategies demand a continuous adaptation and careful selection of a product. Hopefully, completely new modes of action are emerging, as in Glare et al. (2012). A margin of progress that is important in improving bio-insecticides is the formulation design enhancing the establishment of bio-control agents in the roots or leaves of the crop (Kamilova et al., 2005; Bruck, 2010). For example, a seed treatment system with a bio-insecticide could emerge (Kabaluk and Ericsson, 2007). Without talking about genetically modified plants, genetic engineering can also be used to express pesticidal compounds in plants via endophytes (Qi et al., 2011). These new approaches are very promising for the management of bio-pesticides resistance because they have different modes of action. The advantage of working on living organisms (complex systems) unlike chemical molecules lies in the possibilities of tackling the problem from several points of view. Each new progress will lead to the better sustainability of these techniques.

Bio-insecticides are a future solution in substitution for or in addition to synthetic products. However, we must be careful and use them reasonably to avoid failures. A bio-insecticide can be the only solution in some cases of total resistance to chemical products, which is the case for insects that are resistant to pyrethroids that can be treated with fungal entomopathogenic organisms. Having a completely different mode of action, no cross-resistance has been observed. In contrast, cross-resistance can be observed between bio-insecticides and synthetic insecticides with a related mode of action, as is the case between the neonicotinoids and spinosad (Mota-Sanchez et al., 2006).

An increase in the acceptable damage threshold is undoubtedly a key factor if we want to reduce the selection pressure on pests and therefore the risks of resistance outbreaks. This increase requires a change in the market practices (acceptance of imperfect products, short distribution circuit to avoid prolonged conservations) and an increase in the market prices (because of bigger losses).

The use of bio-insecticides therefore requires a good knowledge of their mode of action to alternate targets and thus associates them with conventional plant treatments. However, the acquired knowledge would permit the use of these bio-insecticides wisely and include them in a larger set of resistance management. This knowledge would play a crucial role in integrated pest management. Alternating between chemical families, the use of the previously mentioned preventive methods (cultural, physical, genetic, etc.) associated with bio-insecticides could significantly prevent the occurrence or the development of some resistances.

### Conflict of interest statement

The authors declare that the research was conducted in the absence of any commercial or financial relationships that could be construed as a potential conflict of interest.

## Abbreviations

ALP: alkaline phosphatases
APN: aminopeptidase N
AFP: antifungal protein
ABC transporter: ATP-binding cassette transporter
AcMNPV: *Autographa californica* nucleopolyhedrovirus
Bt: Bacillus thuringiensis
Bti: Bacillus thuringiensis subsp israeliensis
BCA: biocontrol agent
CIs: chymotrypsin inhibitors
CpGV: *Cydia pomonella* granulovirus
GABA: γ-aminobutyric
GMO: genetically modified
GV: granuloviruses
nAChRs: nicotinic acetylcholine receptor
NPV: nucleopolyhedroviruses
APP: P-aminopeptidase
PM: peritrophic membrane
PFT: Pore-Forming Toxins
PhopGV: Phthorimaea operculella granulovirus
PbGV: Pieris brassicae granulovirus
PBO: piperonylbutoxide
PTTH: prothoracicotropic hormone
SPI: serine protein inhibitor
(TIs): trypsin inhibitors.
